# White Adipose Tissue as a Functional Target of Secondary Bile Acids in Rainbow Trout (*Oncorhynchus mykiss*)

**DOI:** 10.1007/s10126-026-10622-5

**Published:** 2026-05-09

**Authors:** Gabriel Pérez-Tierra, Jessica Calo, Ayelén M. Blanco, José L. Soengas

**Affiliations:** Centro de Investigación Mariña, Laboratorio de Fisioloxía Animal, Departamento de Bioloxía Funcional e Ciencias da Saúde, Facultade de Bioloxía, Universidade de Vigo, Vigo, E-36310 Spain

**Keywords:** Secondary bile acids, Adipose tissue, RNAseq, Transcriptome, Glucose and lipid homeostasis, Fish

## Abstract

Secondary bile acids (SBAs) are increasingly recognized as hormone-like signals in vertebrates, yet their regulatory potential in fish adipose tissue remains largely unexplored. This study evaluated the acute effects of SBAs (500 µM lithocholic acid -LCA-, 1500 µM deoxycholic acid -DCA-, and their taurine conjugates: 1000 µM T-LCA and 600 µM T-DCA) on the transcriptomic profile, glucose and lipid homeostasis, and bile acid (BA)–related pathways in rainbow trout white adipose tissue (WAT) six hours after intragastric administration. The main results showed that LCA emerged as the main metabolic modulator, enhancing glycolytic entry points via increased hexokinase and pyruvate kinase activities, modulating energy-sensing genes *prkaa1/2* (Ampk) and *mtor* (mTOR), and promoting coordinated adjustments in lipid metabolism, including upregulation of *acly* and *hadh* (Hoad), increased Acly activity, and reduced NEFA levels suggesting a potential enhancement of metabolic flexibility and intracellular lipid turnover. In contrast, DCA induced a more restricted response, upregulating mRNA abundance of genes encoding the BA transporters Ostα1 and Ostβ, the receptor FXRα-1, and Cyp8b1 isoforms, suggesting a targeted role in local BA sensing and signaling. Taurine-conjugated SBAs generally produced attenuated effects, with T-DCA tending to promote lipid retention and T-LCA evoking minimal changes. Overall, this study provides the first evidence of BA–related elements in rainbow trout WAT and suggests that SBAs can potentially modulate adipose tissue function in a manner dependent on BA identity, laying the foundation for future studies on their physiological and nutritional roles.

**Supplementary Information:**

The online version contains supplementary material available at 10.1007/s10126-026-10622-5.

## Introduction

Bile acids (BAs) have been redefined in mammals from classical emulsifiers of dietary lipids to multifaceted metabolic signals that influence energy balance, nutrient handling, and endocrine communication (Perino et al. 2021). BAs are synthesized in the liver from cholesterol as primary BA, initially in an unconjugated form and subsequently conjugated with glycine or taurine, with a subset of these primary BAs undergoing microbial transformation in the gut to form secondary bile acids (SBAs) (Wahlstrom et al. 2016). These molecules circulate through the enterohepatic pathway, where region-specific transport systems mediate their intestinal uptake, return to the liver, and subsequent reconjugation, thereby sustaining a tightly regulated BA pool (Chiang and Ferrell 2020). Throughout this cycle, BAs transiently reach the gut, liver, and systemic circulation, enabling them to act beyond digestion and participate in metabolic regulation (Lefebvre et al. 2009). These regulatory actions are primarily mediated by two receptors: the nuclear farnesoid X receptor (FXR) and the G-protein–coupled receptor TGR5 (GPBAR1), each displaying distinct ligand preferences and downstream effects (Ridlon et al. 2006). Chenodeoxycholic acid (CDCA) serves as the most potent endogenous FXR agonist, whereas the SBAs lithocholic (LCA) and deoxycholic acids (DCA) constitute the strongest endogenous agonists identified so far for TGR5 (Parks et al. 1999; Kawamata et al. 2003).

Through these receptors, BAs act as hormone-like molecules that coordinate transcriptional and non-genomic responses involved in metabolic homeostasis, with FXR controlling BA synthesis, lipid and glucose metabolism (Lefebvre et al. 2009; Chiang and Ferrell 2020). Its activation, together with the intestinal induction of fibroblast growth factor 19 (FGF19), effectively represses BA synthesis by inhibiting the enzyme cholesterol 7α-hydroxylase (CYP7A1) and the sterol 12-alpha-hydroxylase (CYP8B1) through the small heterodimer partner (SHP)- and FGFR4–β-Klotho–dependent pathways (Inagaki et al. 2005; Kim et al. 2007; Song et al. 2009). In addition, FXR contributes to postprandial glucose control by limiting the expression of gluconeogenic enzymes such as phosphoenolpyruvate carboxykinase (PEPCK) and glucose-6-phosphatase (G6Pase) (Yamagata et al. 2004; Zhang et al. 2006), and exerts a pronounced anti-lipogenic effect through the suppression of the sterol regulatory element-binding protein 1c (SREBP-1c) and the reduction of the fatty acid synthase (FAS) (Watanabe et al. 2004; Ma 2006). In the liver, TGR5 also contribute to metabolic regulation, although its role is more limited than that of FXR and extends predominantly to extrahepatic tissues (Perino et al. 2021). Traditionally, research has focused on BA regulatory effects within their principal target tissues—the liver and the gut; however, emerging evidence indicates that circulating BAs constitute additional and underappreciated regulatory effectors with the capacity to exert endocrine actions in peripheral tissues.

Within this context, white adipose tissue (WAT) has recently emerged as a relevant yet insufficiently characterized target of systemic BAs (Schmid et al. 2023). This paradigm shift has led to the proposal of the term “bilokines” to conceptualize their potential extrahepatic actions, including FXR- and TGR5-mediated regulation of adipocyte differentiation, adipokine and cytokine secretion (Schmid et al. 2019), lipid storage (Van Zutphen et al. 2019; Shinohara and Fujimori 2020), thermogenic activity (Watanabe et al. 2006; Broeders et al. 2015; Velazquez-Villegas et al. 2018; Chen et al. 2023), energy expenditure (Svensson et al. 2013), and insulin signaling and glucose uptake (Rizzo et al. 2006; Axling et al. 2020; Dehondt et al. 2023). For instance, FXR activation promotes lipogenic gene expression and supports adipogenesis (Shinohara and Fujimori 2020), whereas TGR5 activation enhances lipolysis and mitochondrial biogenesis, thereby contributing to beige adipocyte recruitment (Velazquez-Villegas et al. 2018). Furthermore, specific BAs such as CDCA can shift the metabolic profile toward increased oxidative capacity, limiting triglyceride accumulation (Teodoro et al. 2016; Chen et al. 2017). Taken together, although hepatic mechanisms are well established, the growing recognition of direct BA effects in WAT underscores their importance as metabolic regulators in this still underexplored tissue.

In fish, functional knowledge regarding BAs remains limited, despite a growing body of descriptive and functional studies. To date, advances have been made in characterizing BA profiles, fecal loss, and the tissue distribution of genes encoding BA receptors and transporters in rainbow trout (*Oncorhynchus mykiss*) (Murashita et al. 2013, 2014). Studies on dietary BA supplementation in the same species have revealed dose- and lipid-matrix–dependent effects, ranging from improved lipid digestibility to adverse hepatic responses under specific conditions (Amirkolaei et al. 2025), as well as regulatory responses to novel aquafeed ingredients (Murashita et al. 2018; Cocci et al. 2024). Similar variable responses to the dietary supplementation of individual BAs have also been reported in other teleost species, including grass carp (*Ctenopharyngodon idella*; Du et al. 2024) and yellow catfish (*Pelteobagrus fulvidraco*; Zheng et al. 2024). More recently, our own studies in rainbow trout demonstrated SBA reabsorption into the bloodstream and their capacity to differentially modulate the mRNA abundance of receptors, transporters, and regulatory genes involved in BA signaling and feed-intake control—at both gastrointestinal and hypothalamic levels (Pérez-Tierra et al. 2025)—as well as hepatic glucose, lipid and BA metabolism (Pérez-Tierra et al. 2026), highlighting a broader physiological relevance of these compounds in rainbow trout. Collectively, available studies indicate that BA signaling in fish is highly specific to the chemical identity of individual BAs, consistent with observations in mammalian models (Schmid et al. 2023), and that SBAs not only exert local effects but can also act on distant tissues, such as the brain. However, their putative effects on other peripheral tissues, including WAT, remain entirely unexplored in fish.

Therefore, the aim of the present study was: (I) to provide the first characterization of the mRNA abundance of the main BA transporters, receptors, synthesizing enzymes and additional BA-related regulators in rainbow trout WAT; (II) to evaluate the global impact of intragastrically-administered SBAs (LCA, DCA, taurolithocholic acid-T-LCA-, and taurodeoxycholic acid -T-DCA-) in rainbow trout WAT; and (III) to determine the specific effects of these SBAs on the mRNA abundance of BA-related genes, transcript levels and enzymatic activity of key enzymes involved in glucose and lipid metabolism, mRNA abundance of major transcriptional and signaling factors regulating energy metabolism, and on the levels of essential energy metabolites in WAT, using rainbow trout as a teleost model species.

## Materials and Methods

### Fish

Juvenile rainbow trout (average body weight (bw) = 60 ± 20 g) were sourced from a local aquaculture farm in A Estrada (Spain) and acclimated in 100 L tanks at the University of Vigo (Spain). The tanks operated under a continuous flow-through system with aerated, dechlorinated tap water maintained at 15 ± 1 °C, and a controlled photoperiod of 12 h light/12 h dark (lights on at 08:00 h). Fish were fed once daily at 11:00 h (offered ~ 2.5–3.5% bw) using a commercial dry feed (Biomar, Dueñas, Spain) until apparent satiation. The feed had the following proximate composition: 44% crude protein, 2.5% carbohydrates, 21% crude fat, and 17% ash, providing 20.2 MJ kg⁻¹ of gross energy. All experimental procedures complied with the European Union Directive 2010/63/EU and the Spanish Royal Decree 118/2021 on the protection of animals used for scientific purposes. The study protocol was approved by the Ethics Committee of the University of Vigo and authorized by the Regional Government of Galicia (Xunta de Galicia, authorization ES360570181401/23/FUN01/FIS02/JLSF01). All handling and experimental manipulations were conducted by trained personnel holding the required certifications, in a facility licensed by the Xunta de Galicia (REGA ES360570181401).

### Experimental Design

Fish were distributed into five tanks (*n* = 8 fish per tank) and acclimated for 14 days under the feeding conditions previously described. On the day of the trial, fish were fasted for 24 h, anesthetized with 2-phenoxyethanol (0.02% v/v), individually weighed, and intragastrically administered 1 mL·100 g⁻¹ body weight of distilled water containing 1% DMSO alone (control) or containing one of the tested SBAs: 500 µM LCA, 1500 µM DCA, 1000 µM T-LCA, or 600 µM T-DCA. Doses were determined based on a previous own study (Pérez-Tierra et al. 2025). All compounds were obtained from Sigma (Sigma Chemical Co., St. Louis, MO, USA; catalog numbers: LCA L6250; DCA 30960; T-LCA T7515; T-DCA 580221). Administrations were performed using a blunt-ended syringe fitted with a 13-cm cannula, following established procedures (Calo et al. 2023). Animals were visually monitored for signs of regurgitation during administration, and none were observed. After treatment, fish were returned to their tanks for 6 h. Individuals were then re-anesthetized and euthanized for visceral adipose tissue collection. Fixed treatment doses and sampling time point was based on plasma detection of the same circulating SBAs at 2 h and peripheral tissue responses at 6 h after intragastric administration in the same species (Pérez-Tierra et al. 2025, 2026).

A complementary experiment was conducted to characterize the mRNA abundance of key BA–responsive elements in WAT. Eight 24 h feed-deprived fish were anesthetized with 2-phenoxyethanol (0.02% v/v) and euthanized to collect WAT samples. The transcripts examined included the main BA receptors (*fxra*,* fxrb* and *gpbar1*), their associated FXR-mediated signaling components (*shp* and the hepatocyte nuclear factor-4 alpha-*hnf4a-*), the principal BA transporters (Na^+^ taurocholate cotransporting polypeptide-*slc10a1-*, apical sodium-dependent BA transporter-*slc10a2-*, organic solute transporter alpha-*slc51a*- and beta *-sl51b-* and bile salt export pump-*bsep-*), and the rate-limiting enzymes involved in BA biosynthesis (*cyp7a1*and *cyp8b1*).

### Transcriptomic Analysis

Total RNA was isolated from WAT samples (*n* = 8 per group) using Trizol reagent (Life Technologies, Grand Island, NY, USA) according to the manufacturer’s protocol. RNA aliquots from the same preparations were then shipped on dry ice to BMKGene (Biomarker Technologies; Germany), while the remaining RNA was retained for subsequent qPCR analyses. The purity and integrity of the extracted RNA was confirmed by measuring the optical density (OD) ratio at 260/280 nm with a NanoDrop 2000c spectrophotometer (Thermo, Vantaa, Finland) and an Agilent 2100 Bioanalyzer (Agilent Technologies, CA, USA), respectively. For RNA-seq analysis, samples were selected based on predefined RNA quality criteria to ensure technical reliability. Specifically, the three samples per group with the highest RNA Integrity Number (RIN > 5) out of the total available samples (*n* = 8 per group) were selected for library preparation.

A total of 1 µg of RNA per sample was used as input for library construction. Sequencing libraries were prepared using the NEBNext Ultra™ RNA Library Prep Kit for Illumina (NEB, USA) following the manufacturer’s protocol. Briefly, mRNA was isolated from total RNA using poly-T oligo-conjugated magnetic beads. Fragmentation was performed in the NEBNext First Strand Synthesis Reaction Buffer (5×) under high-temperature conditions in the presence of divalent cations. First-strand cDNA synthesis was carried out with random hexamer primers and M-MuLV reverse transcriptase, followed by second-strand synthesis using DNA Polymerase I and RNase H. Overhangs were subsequently converted into blunt ends through exonuclease and polymerase activities. After 3′-end adenylation, NEBNext adaptors containing a hairpin loop structure were ligated to the DNA fragments to enable hybridization. Library fragments of approximately 240 bp were selected using the AMPure XP system (Beckman Coulter, USA). Size-selected, adaptor-ligated cDNA was then treated with 3 µL of USER enzyme (NEB, USA) at 37 °C for 15 min followed by 95 °C for 5 min prior to PCR amplification. PCR enrichment was performed using Phusion High-Fidelity DNA polymerase, universal PCR primers, and index primers. The resulting PCR products were purified with the AMPure XP system, and library quality was evaluated using the Agilent 2100 Bioanalyzer. Then, cluster generation of the indexed libraries was performed on a cBot Cluster Generation System using the TruSeq PE Cluster Kit v4-cBot-HS (Illumina), according to the manufacturer’s instructions. Sequencing was subsequently carried out on an Illumina platform to produce paired end reads. To analyze the quality control, raw reads in FASTQ format were processed using in-house Perl scripts. Clean reads were obtained by removing adapter-containing reads, reads with poly-N, and reads with low base quality. Quality metrics including Q20, Q30, GC content, and sequence duplication levels were calculated. All downstream analyses were conducted using high-quality clean reads. Two FASTQ files were obtained for each sample containing sequences measured from both ends.

For comparative analysis, adapter sequences and low-quality reads were removed prior to alignment. Clean reads were mapped to the *O. mykiss* reference genome (GCF_013265735.2_USDA_OmykA_1.1) using HISAT2. Only reads with either a perfect match or a single mismatch were retained for subsequent analyses and annotation. Gene expression levels were quantified as fragments per kilobase of transcript per million mapped fragments (FPKM). Reproducibility among biological replicates was assessed using Pearson’s correlation coefficient (R) on expression data transformed as log₂ (FPKM + 1). Differential expression analysis between the control group and each treatment was conducted using DESeq2. Resulting P-values were adjusted using the Benjamini–Hochberg procedure to control the false discovery rate (FDR). Genes with a fold change (FC) ≥ 1.5 and an adjusted P-value < 0.01 were considered differentially expressed. Gene Ontology (GO) enrichment of the differentially expressed genes (DEGs) was carried out using the GOseq R package, which applies the Wallenius non-central hypergeometric model to correct for gene length bias. KEGG pathway enrichment analysis was performed with KOBAS to identify statistically overrepresented metabolic and signaling pathways. All transcriptomic analyses were conducted through the BMKCloud platform (www.biocloud.net).

### Quantification of mRNA Abundance by qPCR

RNA from WAT samples of the main experiment (*n* = 8 per group) was processed as described above. Separately, total RNA was extracted from an independent set of WAT samples used to characterize the mRNA abundance of BA-related elements in the complementary experiment (*n* = 8) using Trizol reagent. The purity of the RNA was assessed by determining the 260/280 nm absorbance ratio using a NanoVue™ Plus spectrophotometer (GE Healthcare, Chicago, IL, USA). RNA samples from the main and the complementary experiment were subsequently treated with RQ1-DNase following the guidelines provided by the manufacturer. Then, 2 µg of RNA were reverse transcribed into complementary DNA (cDNA) in a 20 µL reaction using the GoScript™ Reverse Transcription Mix with Random Primers (Promega, Madison, WI, USA), according to the supplied protocol. Quantitative real-time PCR (RT-qPCR) was performed on a CFX Connect Real-Time PCR system (Bio-Rad, Hercules, CA, USA) using MAXIMA SYBR Green qPCR Master Mix (Life Technologies). Reactions were assembled in 96-well plates, with each 10 µL reaction containing 2 µL of cDNA (prediluted 1:8) and 500 nM of each primer. All samples were analyzed in duplicate. Thermal cycling consisted of an initial denaturation step at 95 °C for 10 min, followed by 40 cycles of 95 °C for 15 s and gene-specific annealing/extension at the temperatures shown in Table 1 for 30 s. Specificity of amplification was confirmed by melting-curve analysis, performed by increasing the temperature from 70 to 90 °C in 0.5 °C increments every 5 s, yielding a single peak for each reaction. Relative mRNA levels were calculated using the 2^–ΔΔCt^ method. The genes *actb* (β-actin) and *eef1a1* (elongation factor 1α) were used as reference genes due to their stable expression. Primer sequences analyzed are listed in Table 1. Their amplification efficiency was subsequently verified to fall within the acceptable range (90–110%).

**Table 1:** Nucleotide sequences of primers used to determine mRNA abundance of target transcripts with their GenBank accession number and annealing temperature (T)

Transcript	Forward primer	Reverse primer	Accession number	T (°C)
*acac1*	TTGTTACCCGCTTTGGTGGT	TACTCTGCGTTGGCCTTCAG	XM_036990264.1	60
*acac2*	TGAGGGCGTTTTCACTATCC	CTCGATCTCCCTCTCCACT	XM_036935547.1	60
*acly*	CTGAAGCCCAGACAAGGAAG	CAGATTGGAGGCCAAGATGT	XM_036955916.1	60
*actb*	GATGGGCCAGAAAGACAGCTA	TCGTCCCAGTTGGTGACGAT	NM_001124235.1	60
*bsep*	CGGCTTCGCCCAGTGTGTCG	CCCAGCGCTGTGCCACTGGT	NM_001124656.1	62
*cpt1b*	GATGTTCCGTGAGGGTAGGA	TTGTCTTGCATGGCTCTGAC	NM_001171855.1	60
*cyp7a1-1*	CGTCTGTCGAGCGCATCAAT	TTGTCTTCTCCTGGCCGTTGT	AB675933.1	60
*cyp7a1-2*	TTCCTGTGCGACCCTTTCTC	AGGTCTGGTGGAGGTTCTCT	AB675934.1	60
*cyp8b1-1*	CACCAAATTCGCAGAGCAGC	CAGGCCATCCTCATTCCAGG	AB675935.1	60
*cyp8b1-2*	GTTACCTGGCTCTGTTCGGT	CCTCTCCGCCTCTCTCTTCT	AB675936.1	60
*eefla1*	GTCTACAAAATCGGCGGTAT	CTTGACGGACACGTTCTTGA	AF498320	60
*fasn*	GAGACCTAGTGGAGGCTGTC	TCTTGTTGATGGTGAGCTGT	XM_036956054.1	60
*fbp1*	GCTGGACCCTTCCATCGG	CGACATAACGCCCACCATAGG	AF333188	60
*fxra1*	ACCCCCAATACAGCGTTGAG	GTACCCAGATGCCTTGTCCC	XM_021616163.2	62
*fxra2*	TTCTCTGTCTACGGACCGAC	TGGTCCTCTTTACCAGGGACA	XM_021566428.2	60
*fxrb1*	CCTGCGTAAATGTCGTGCTG	GCCGAAGAGCCTCCACTATC	AB675939.1	60
*fxrb2*	GGCTGCAAAGGTTTCTTCCG	CTCCTCTGTGTTTGGTCCCC	XM_021574106.2	62
*g6pc*	CTAGGCGTGGACCTGCTATG	GTCCTAAAGAGGGTCGTGCC	AB889398.1	60
*gpbar1*	GAACACAACCACCGCAACTG	CCACTAGCAGGCTCAGGAAG	XM_021570747.2	62
*hadh*	ATCCGCCAAGGGAATAGAAGG	TGGCGAAAATGGTGTGTGC	XM_021573383.2	60
*hnf4a*	TCCAATTCCCTCTCCTCCCA	GTTTGGTGATGGTTGGCTGC	XM_021565992.2	60
*lpl*	TAATTGGCTGCAGAAAACAC	CGTCAGCAAACTCAAAGGT	AJ224693	60
*mlxipl*	GTAGCAACCAAAGGGCACAGG	GCTGCAGCTGAGATCCAGACA	MG310162	60
*mtor*	ATGGTTCGATCACTGGTCATCA	TCCACTCTTGCCACAGAGAC	XM_021615845.2	60
*nr1h3*	TGCAGCAGCCGTATGTGGA	GCGGCGGGAGCTTCTTGTC	FJ470291	60
*pck*	CTACCTAGCCCACTGGCTGA	GGGCGTTGTCACCGAAAC	NM_001124275.1	60
*pfk*	GGTGGAGATGCACAAGGAAT	CTTGATGTTGTCCCCTCCAT	XM_036959534.1	60
*pklr*	GGGAGACGGCTAAAGGACTG	AGCTCCAGCACAGCATTTGA	XM_036940182.1	62
*pparab*	CAGTGCACCTCCGTAGAGAC	ACAGTGCCTCATACACGCC	NM_001197211.1	60
*pparg*	GACGGCGGGTCAGTACTTTA	ATGCTCTTGGCGAACTCTGT	NM_001197212.1	60
*prkaa1*	ATCTTCTTCACGCCCCAGTA	GGGAGCTCATCTTTGAACCA	XM_021590586.2	60
*prkaa2*	GGGCTACCATTAAAGACATTAGGG	ACTCGGTGCTCTCAAACTTG	XM_036968699.1	60
*shp-1*	AGTCAAGCCACAACAACCTGA	TTTCACCAGCACTCCCGATG	XM_021562336.2	62
*shp-2*	CAAGCCACAACAACCCGAAC	TGGAGGAGCGACAGTTGATC	XM_021595923.2	62
*slc10a1*	CTCCCCGGTTATGGACATGG	TGAAGGCAGTGAGAGGCATG	AB675930.1	62
*slc10a2*	AGGCCGTCGTCATCATCATC	AGGCTGTCATGCTGATACTGAG	AB675929.1	62
*slc51a1*	GATCGTTCCAGTTTGCCGTG	CAGATGGCTGCTCCCGTTAT	AB675931.1	60
*slc51a2*	ATCATCTGGGTCAATGGGGC	AGGCGGAGGTCATGTCAGTA	AB675932.1	60
*slc51b*	GGAGGCGGAAGAACTACAGG	CGTCATTCCAGGTCACCACA	XM_036963170.1	62
*srebp1*	GACAAGGTGGTCCAGTTGCT	CACACGTTAGTCCGCATCAC	KP342261.1	60

The first column indicates genes encoding for: *acac1*, acetyl-CoA carboxylase; *acac2*, acetyl-CoA carboxylase 2; *acly*, ATP citrate synthase; *actb*, β-actin; *bsep*, bile salt export pump; *cpt1b*, carnitine palmitoyltransferase 1B; *cyp7a1-1*, cholesterol 7α-hydroxylase 1–1; *cyp7a1-2*, cholesterol 7α-hydroxylase 1–2; *cyp8b1-1*, sterol 12-alpha-hydroxylase 1–1; *cyp8b1-2*, sterol 12-alpha-hydroxylase 1–2; *eefla1*, elongation factor-1α; *fasn*, fatty acid synthase; *fbp1*, fructose 1,6-bisphosphatase; *fxra1*, farnesoid X receptor like-α1; *fxra2*, farnesoid X receptor like-α2; *fxrb1*, farnesoid X receptor like-β1; *fxrb2*, farnesoid X receptor like-β2; *g6pc*, glucose-6-phosphatase; *gpbar1*, G protein-coupled bile acid receptor; *hadh*, 3-hydroxyacyl-CoA dehydrogenase; *hnf4a*, hepatocyte nuclear factor-4 alpha; *lpl*, lipoprotein lipase; *mlxipl*, carbohydrate-responsive element-binding protein; *mtor*, mechanistic target of rapamycin; *nr1h3*, liver X receptor alpha; *pck*, phosphoenolpyruvate carboxykinase; *pfk*, 6-phosphofructo 1-kinase; *pklr*, pyruvate kinase; *pparab*, peroxisome proliferator-activated receptor alpha b; *pparg*, peroxisome proliferator-activated receptor gamma; *prkaa1*, AMP-activated protein kinase alpha-1; *prkaa2*, AMP-activated protein kinase alpha-2; *shp-1*, small heterodimer partner-1; *shp-2*, small heterodimer partner-2; *slc10a1*, Na^+^ taurocholate cotransporting polypeptide; *slc10a2*, apical sodium-dependent bile acid transporter; *slc51a1*, organic solute transporter alpha-1; *slc51a2*, organic solute transporter alpha-2; *slc51b*, organic solute transporter beta; *srebp1*, sterol regulatory element-binding protein 1

### Assessment of Specific Enzyme Activities

WAT samples for enzymatic activity analysis (*n* = 8) were homogenized in five volumes of lysis buffer (250mM sucrose, 50 mM Trizma Base, 1 mM EDTA, 1 mM dithiothreitol, and 1.02 mg·mL⁻¹ protease inhibitor cocktail; Sigma). Homogenates were centrifuged at 3000 rpm for 10 min at 4 °C. The supernatants were collected, re-centrifuged at 12,000 g for 15 min at 4 °C and the final supernatants were used for the assays.

Enzyme activities were measured using an INFINITE 200 Pro microplate reader (Tecan, Männedorf, Switzerland). For most enzymes, activity was quantified by monitoring changes in NADH or NADPH absorbance at 340 nm, whereas carnitine palmitoyltransferase 1 (Cpt-1) activity was assessed by detecting the formation of the 5,5′-dithiobis-(2-nitrobenzoic acid)-CoA complex at 412 nm. Reactions were initiated by adding 15 µL of supernatant—previously adjusted to a standardized protein concentration—while control wells were prepared identically but without substrate. Final reaction volumes ranged from 265 to 295 µL, and incubations were performed at 37 °C for periods between 10- and 120-min. Maximal reaction rates were used for activity calculations, based on preliminary assays establishing optimal substrate concentrations. Activities of total hexokinase [Hk; EC 2.7.1.1], pyruvate kinase [Pk; EC 2.7.1.40], fructose-1,6-bisphosphatase [Fbpase; EC 1.1.1.49], ATP citrate lyase [Acly; EC 2.3.3.8], Cpt-1 [EC 2.3.1.21] and 3-hydroxyacyl-CoA dehydrogenase [Hoad; EC 1.1.1.35] were determined following previously established protocols (Conde-Sieira et al. 2020; Talarico et al. 2025; Comesaña et al. 2025). Enzymatic activities were normalized to total protein content to obtain specific activity values. Protein concentration was quantified using the bicinchoninic acid (BCA) assay, with bovine serum albumin (BSA; Sigma) as the standard.

### Analysis of Metabolite Levels

WAT samples for metabolite analysis (*n* = 8) were homogenized in 1.5 volumes of 0.6 M perchloric acid. After homogenization, samples were neutralized with 1.5 volumes of 1 M potassium bicarbonate and centrifuged at 13,500 rpm for 4 min at 4 °C. The resulting supernatants and the remaining pellet (previously resuspended in 1 mL of isopropanol) were used for metabolite determinations. Glucose and the soluble fraction of triglycerides, cholesterol, and non-esterified fatty acids (NEFAs) were quantified in the supernatants using commercial assay kits (Spinreact, Barcelona, Spain for glucose and triglycerides; Wako Chemicals, Neuss, Germany for NEFAs). The resuspended pellet was used to determine the residual fraction of triglycerides, cholesterol, and NEFAs using the corresponding commercial kits. Total metabolite content was determined from the combined supernatant and pellet fractions. All samples were analyzed in duplicate.

### Statistical Analysis

Outliers within each group were identified using Grubbs’ test (*p* < 0.05) and removed. Normality was assessed within each group using the Shapiro–Wilk test, and non-normally distributed data were log-transformed prior to further analysis. Homogeneity of variances was evaluated prior to statistical comparisons using Levene’s test. Group comparisons were performed using two-tailed, unpaired Student’s t-tests when variances were equal (*p* ≥ 0.05), or with the Welch correction otherwise. To account for multiple testing, p-values were adjusted using the Benjamini–Hochberg false discovery rate (FDR) procedure applied independently to each analytical dataset. Specifically, adjustments were performed separately within each experimental assay to maintain the independence of the statistical evaluations within each dataset. Adjusted p-values (*P*_adj_) below 0.05 were considered statistically significant. Statistical analyses were performed in RStudio 4.1.2, and figures were generated with GraphPad Prism 8.0.1.

## Results

### Differential Gene Expression in Rainbow Trout White Adipose Tissue Following SBA Administration

**Fig. 1:**
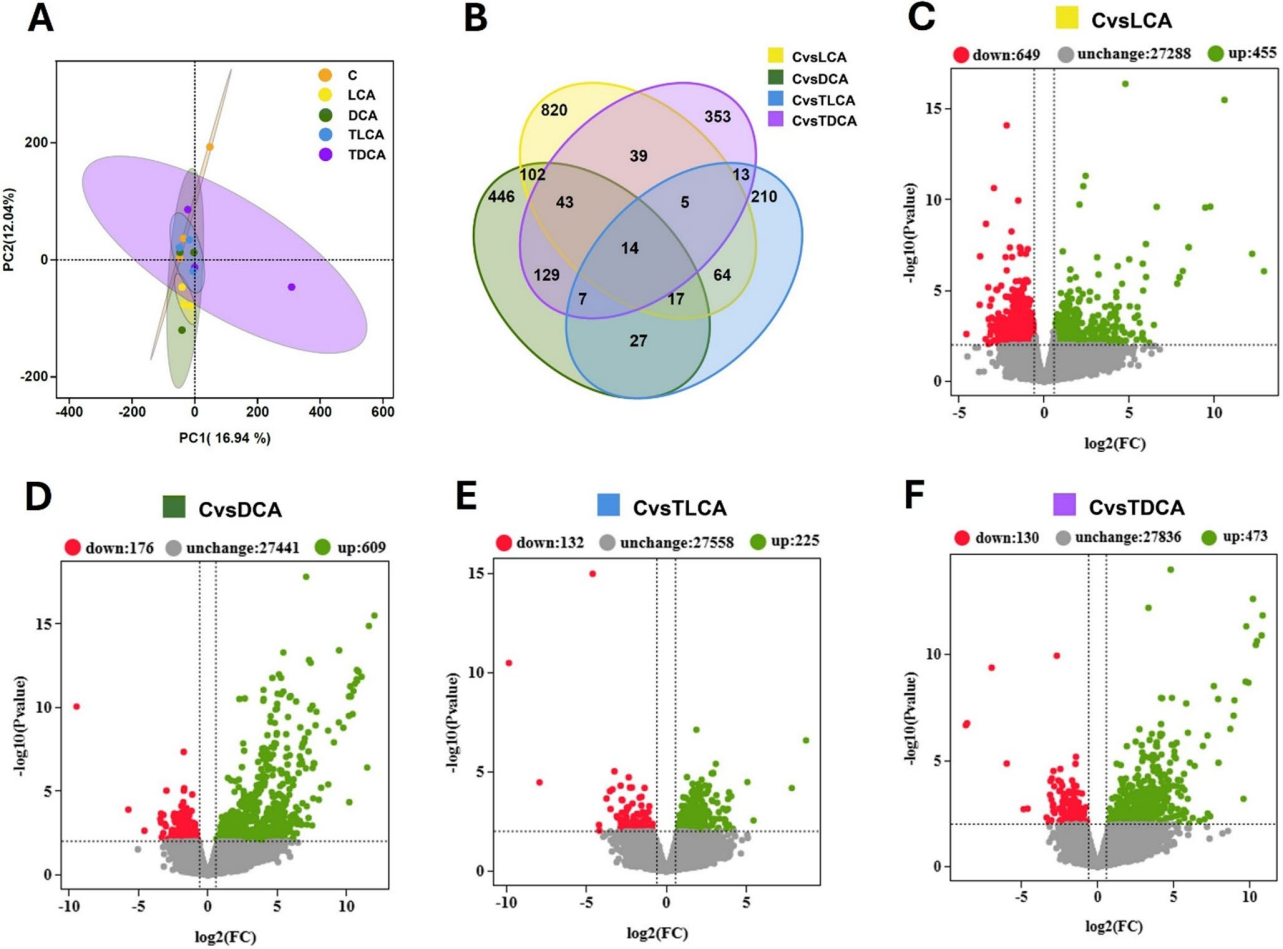
Transcriptomic profiling by RNA-seq of white adipose tissue (WAT) in rainbow trout intragastrically administered with SBAs: 500 µM lithocholic acid (LCA), 1500 µM deoxycholic acid (DCA), 1000 µM taurolithocholic acid (T-LCA) or 600 µM taurodeoxycholic acid (T-DCA). (**A**) Principal component analysis (PCA) of control and treatment groups. (**B**) Venn diagrams showing the overlap of upregulated and downregulated genes between control and each treatment. (**C–F**) Volcano plots illustrating differentially expressed genes for the following comparisons: control vs. LCA (**C**), control vs. DCA (**D**), control vs. T-LCA (**E**) and control vs. T-DCA (**F**). FC: fold change

A total of 104.06 Gb high-quality paired-end reads were obtained from the 15 libraries (3 per treatment) and more than 94.37% of bases in each sample had a Q-score no less than Q30 (Supplementary Table S1). Pearson’s correlation coefficients among biological replicates within each experimental group exceeded 0.84 (Supplementary Table S2) and the mapping ratio of each sample against reference genome ranged from 89.46% to 91.70% (Supplementary Table S1). Principal component analysis (PCA) showed a partial overlap between the control and SBA-treated groups. Intra-group variability was generally low across most treatments, whereas a comparatively higher dispersion was detected within the T-DCA group (Fig. 1A). Based on the reference genome of *Oncorhynchus mykiss*, 6,652 genes were optimized and 8,543 novel genes were discovered. The Venn diagram (Fig. 1B) illustrates the comparative analysis of differentially expressed genes (DEGs) between the control and each treatment condition. Relative to the control group, LCA, DCA, T-LCA, and T-DCA treatments resulted in 1104, 785, 357, and 603 DEGs, respectively. Among these, LCA, DCA, T-LCA, and T-DCA treatments resulted in distinct patterns of gene regulation, with 649/455, 176/609, 132/225, and 130/473 genes being down- and up-regulated, respectively, as shown in each corresponding volcano plot (Fig. 1C–F). The remaining genes showed no significant changes in expression, comprising 27,288 genes for LCA, 27,441 for DCA, 27,558 for T-LCA, and 27,836 for T-DCA.

**Fig. 2:**
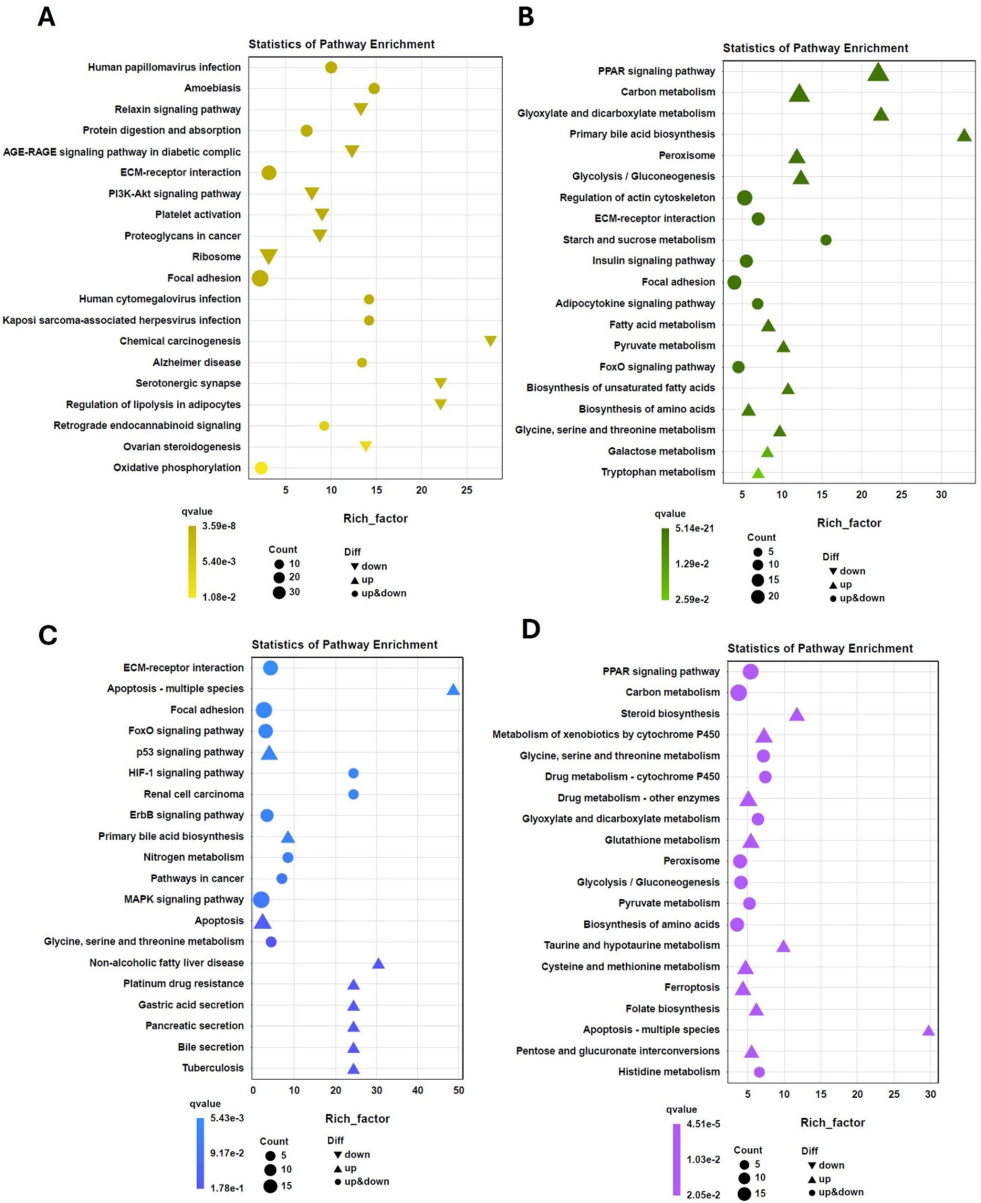
Transcriptomic profiling of bile acid–induced pathway enrichment and bile acid–related gene regulation in rainbow trout white adipose tissue (WAT) following SBA administration. (**A–D**) Top 20 KEGG pathways enriched among up- and down-regulated differentially expressed genes (DEGs) in control fish compared with those treated with LCA (**A**), DCA (**B**), T-LCA (**C**) and T-DCA (**D**). Samples correspond to WAT collected 6 h after intragastric administration of 1 mL·100 g⁻¹ body weight of either distilled water with 1% DMSO alone (control) or containing 500 µM lithocholic acid (LCA), 1500 µM deoxycholic acid (DCA), 1000 µM taurolithocholic acid (T-LCA) or 600 µM taurodeoxycholic acid (T-DCA)

KEGG pathway analysis identified 194, 180, 108, and 162 pathways significantly affected by LCA, DCA, T-LCA, and T-DCA treatments, respectively (Supplementary Table S3). In parallel, Gene Ontology (GO) enrichment analysis—categorized into biological process, cellular component, and molecular function—revealed 35, 32, 31, and 34 enriched categories upon treatment with LCA, DCA, T-LCA, and T-DCA, respectively (Supplementary Table S4). The top 20 enriched KEGG pathways for DEGs between the control and each treatment group are shown in Fig. 2. Overall, each treatment induced distinct global transcriptional responses. In the LCA group (Fig. 2A), regulation of lipolysis in adipocytes emerged as the most significantly enriched lipid metabolism–related pathway, while the predominantly enriched pathway was chemical carcinogenesis. Notably, a substantial proportion of LCA-responsive DEGs were associated with cellular structure and organization, including terms related to cellular integrity, intracellular components, binding elements, and fundamental cellular processes (Supplemental Fig. S1).Within these categories, LCA treatment was characterized by a high number of DEGs predominantly downregulated, whereas the remaining treatments exhibited an opposite pattern, with a greater proportion of upregulated DEGs across the same functional categories (Supplemental Fig. S1). Compared to LCA, DCA exerted a much stronger effect on metabolic pathways (Fig. 2B), with primary BA biosynthesis emerging as the most significantly enriched pathway, followed by PPAR signaling, fatty acid metabolism, and biosynthesis of unsaturated fatty acids, all of which are associated with lipid homeostasis, as well as glycolysis/gluconeogenesis, related to glucose homeostasis. Consistently, among the top 20 enriched pathways, T-LCA was characterized by enrichment of primary BA biosynthesis and bile secretion (Fig. 2C), whereas T-DCA predominantly enriched PPAR signaling and glycolysis/gluconeogenesis pathways (Fig. 2D). Notably, apoptosis emerged as a predominantly enriched pathway in both taurine-conjugated treatments.

**Fig. 3:**
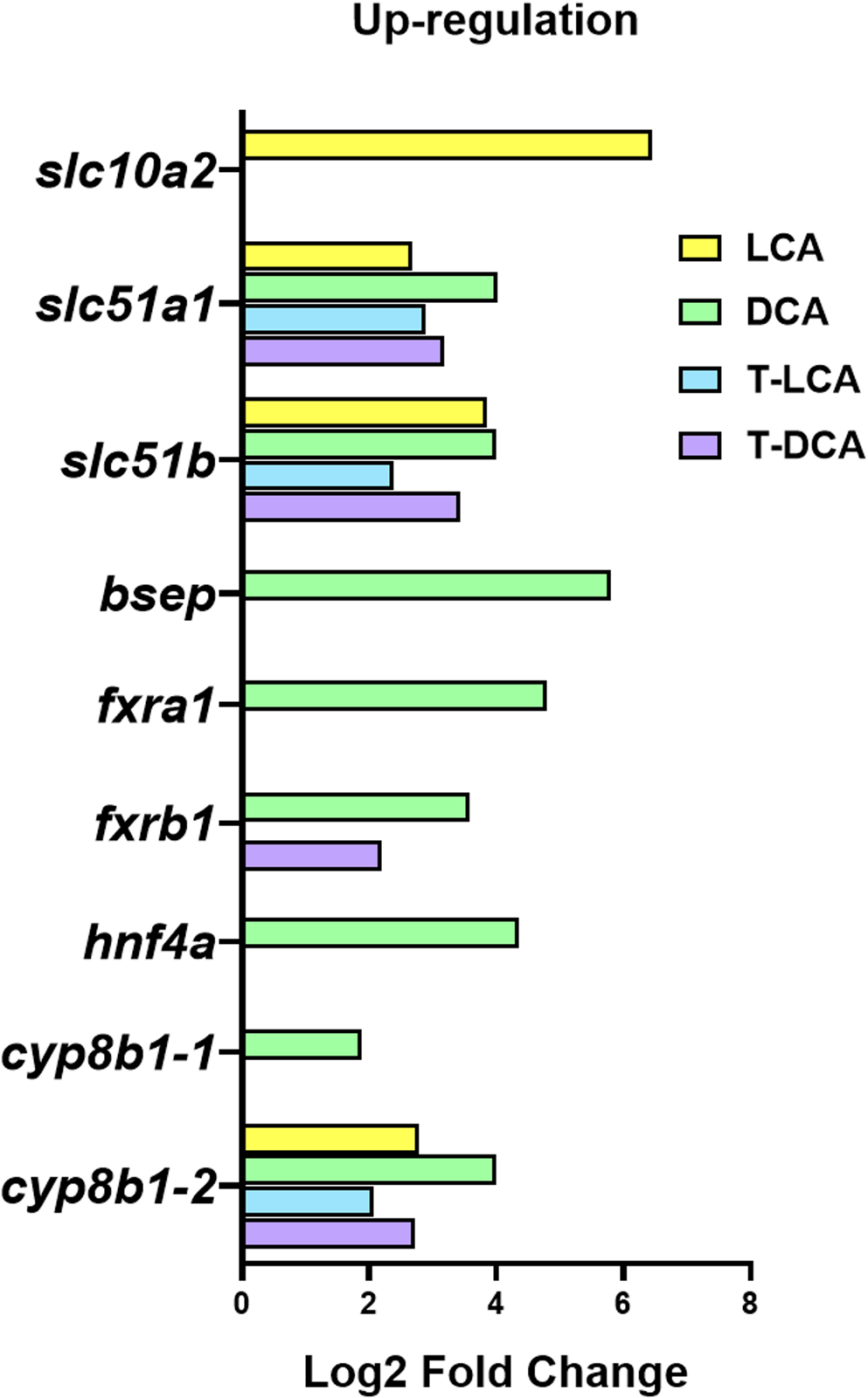
Histogram represents the distribution of DESeq2-derived log_2_ fold change (log_2_FC) values for differentially expressed genes (DEGs) involved in bile acid metabolism in white adipose tissue (WAT), including *slc10a2*, *slc51a1*, *slc51b*, *bsep*, *fxra1*, *fxrb1*, *hnf4a*, *cyp8b1-1* and *cyp8b1-2*. Log_2_FC values are model-based effect size estimates generated from raw RNA-seq counts across biological replicates using DESeq2 and DEGs were defined using an adjusted significance threshold of P_adj_ < 0.01 and FC ≥ 1.5. Samples correspond to WAT collected 6 h after intragastric administration of 1 mL·100 g⁻¹ body weight of either distilled water with 1% DMSO alone (control) or containing 500 µM lithocholic acid (LCA), 1500 µM deoxycholic acid (DCA), 1000 µM taurolithocholic acid (T-LCA) or 600 µM taurodeoxycholic acid (T-DCA)

Based on DESeq2-derived log_2_FC estimates obtained from raw RNA-seq counts across biological replicates (Fig. 3), a focused examination of BA transporters revealed that all BAs tested increased the levels of transcripts encoding Ostα (*slc51a1*) and Ostβ (*slc51b*). In contrast, DCA selectively induced *bsep* expression, whereas LCA specifically upregulated Asbt (*slc10a2*). Concerning BA receptors and Fxr-mediated signaling components, only DCA significantly upregulated *fxra1*, *fxrb1*, and *hnf4a*, while T-DCA specifically induced *fxrb1*. Finally, all BA treatments increased the transcript levels of *cyp8b1-2*, the rate-limiting enzyme involved in BA biosynthesis, whereas only DCA upregulated the *cyp8b1-1* transcript.

### Transcript Levels of Key BA–Related Elements in Rainbow Trout WAT Under Basal Conditions and Following SBA Intragastric Administration

**Fig. 4:**
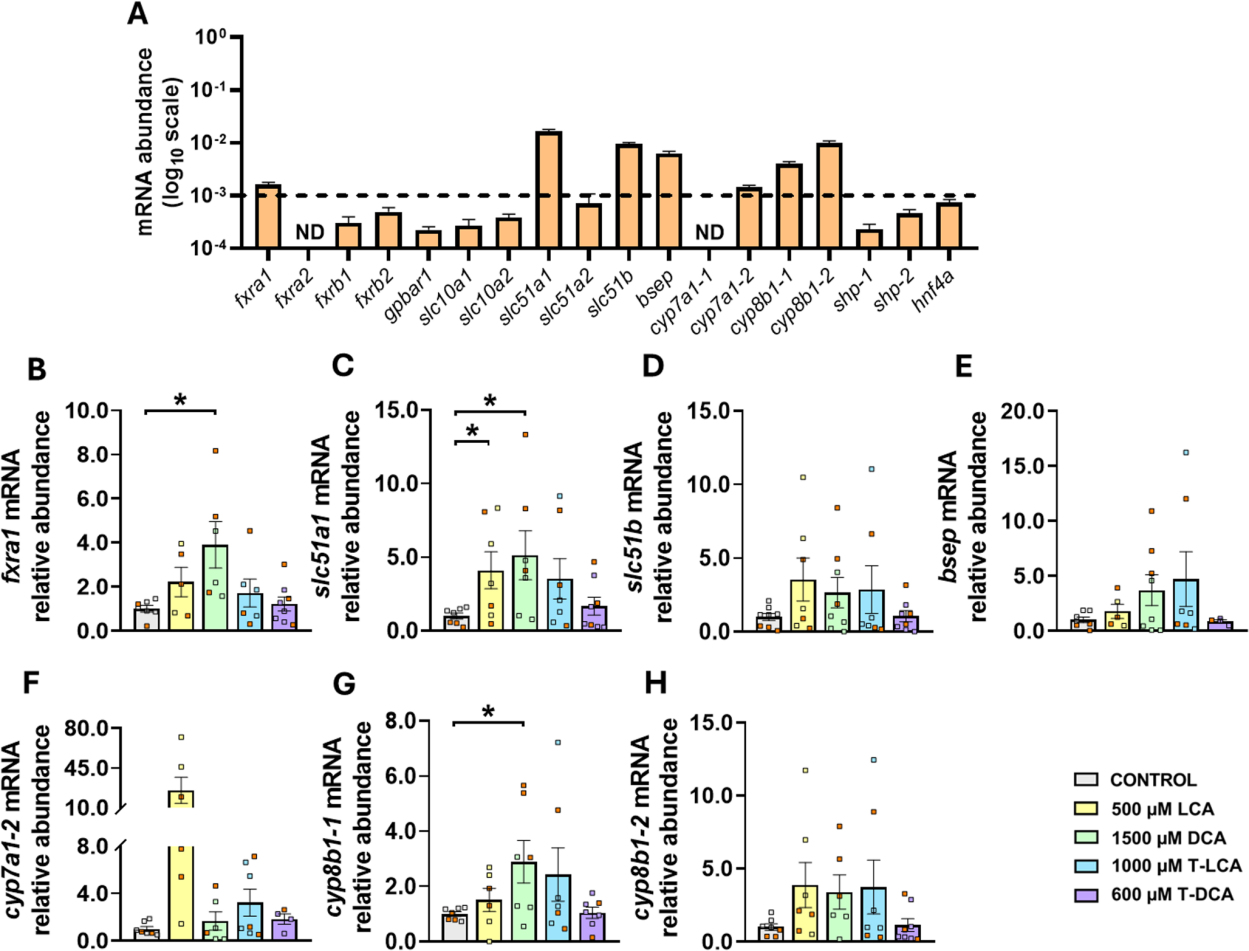
mRNA abundance of key elements of bile acid (BA) metabolism in rainbow trout white adipose tissue (WAT). (**A**) mRNA levels of genes encoding the BA receptors Fxr-α (*fxra1*,* fxra2*), Fxr-β (*fxrb1*,* fxrb2*) and Tgr5 *(gpbar1*), the BA transporters Ntcp (*slc10a1*), Asbt (*slc10a2*), Ost-α (*slc51a1*,* slc51a2*), Ost-β (*slc51b*), and Bsep (*bsep*), the major BA-synthesizing enzymes Cyp7a1 (*cyp7a1-1*,* cyp7a1-2*) and Cyp8b1 (*cyp8b1-1*,* cyp8b1-2*), and other BA-related factors including Shp (*shp-1*,* shp-2*) and Hnf4-α (*hnf4a*), were quantified in 24 h feed-deprived rainbow trout WAT. RT-qPCR data were normalized to *actb* and *eef1a1* and are presented as mean ± SEM (*n* = 8). The y-axis is presented on a log_10_ scale (log10-transformed values) to facilitate visualization of the wide dynamic range in transcript abundance. The dashed line indicates the efficient quantification limit by RT-qPCR. ND: not detected. (B–H) Relative mRNA levels of *fxra1* (**B**), *slc51a1* (**C**), *slc51b* (**D**), *bsep* (**E**), *cyp7a1-2* (**F**), *cyp8b1-1* (**G**) and *cyp8b1-2* (**H**) were measured in WAT of rainbow trout 6 h after intragastric administration of 1 mL·100 g⁻¹ body weight of distilled water with either 1% DMSO alone (control) or containing 500 µM lithocholic acid (LCA), 1500 µM deoxycholic acid (DCA), 1000 µM taurolithocholic acid (T-LCA) or 600 µM taurodeoxycholic acid (T-DCA). RT-qPCR values were normalized to *actb* and *eef1a1* and expressed relative to the control group. Data are shown as mean ± SEM (*n* = 8) and statistical differences between treatments and control were evaluated using Student’s t-test and adjusted using the false discovery rate; *P_adj_ < 0.05. Orange squares in each group indicate the samples originally used for RNA sequencing, which were subsequently integrated into the dataset alongside the remaining samples

The abundance of mRNAs encoding the main BA receptors, transporters, synthesizing enzymes, and other BA-related elements in rainbow trout WAT under basal conditions was evaluated by RT-qPCR (Fig. 4A). Among the *fxr* paralogs, *fxra1* exhibited the highest mRNA abundance, whereas transcripts encoding the remaining isoforms, including the alternative BA receptor Tgr5 (*gpbar1*), were detected at very low levels. Regarding BA transporters, genes encoding Ost-α1 (*slc51a1*), Ost-β (*slc51b*), and Bsep (*bsep*) showed the highest levels, while Asbt (*slc10a2*), Ntcp (*slc10a1*), and Ost-α2 (*slc51a2*) displayed very limited transcript abundance. Finally, both *cyp8b1* variants showed higher transcript levels than the main BA-synthesizing enzyme *cyp7a1*, whereas two key elements involved in functional BA signaling, Shp and Hnf4, exhibited low mRNA abundance.

The mRNA abundance of genes encoding key BA receptors, transporters, and synthesizing enzymes in the rainbow trout WAT 6 h after SBA intragastric administration is shown in Fig. 4 (B-H). Among the evaluated treatments, DCA evoked the strongest transcriptional response, significantly increasing the mRNA abundance of *fxra1* (Fig. 4B), *slc51a1* (Fig. 4C) and *cyp8b1-1* (Fig. 4G), in accordance with the transcriptomic analysis. LCA also triggered specific changes, notably a significant upregulation of *slc51a1* (Fig. 4C). In contrast, no significant differences relative to the control were detected for *slc51b*, *bsep*, *cyp7a1-2* or *cyp8b1-2* (Figs. 4D, E, F, H, respectively). It is worth noting that several genes of interest—*slc10a2*, *fxrb1*, and *hnf4a*—could not be reliably quantified due to their low mRNA abundance, which did not surpass the detection threshold under basal conditions (Fig. 4A). Although SBA treatments can enhance their transcript levels, as suggested in Fig. 3, this independent validation method remains unable to confirm such responses because transcript levels in control animals stay near the quantification limit, mirroring their basal levels. Consequently, despite being measurable under SBA stimulation, these genes cannot be statistically evaluated against the control group.

### SBAs Regulate Energy Metabolism–Related Pathways in Rainbow Trout WAT

Table 2. summarizes the relative mRNA abundance of genes involved in glucose and lipid metabolism, as well as energy-sensing pathways in WAT upon SBA treatment. Relative to the control group, LCA treatment induced a significant upregulation of energy sensors *prkaa1*, *prkaa2*, and *mtor*, whereas DCA exposure selectively increased the transcript levels of glycolytic and gluconeogenic-related genes, including *pfk*, *pklr*, and *fbp1*. No significant transcriptional responses were observed following T-LCA or T-DCA treatments. The mRNA abundance of *pck*, *mlxipl*, and *g6pc* remained unchanged across all experimental conditions. Regarding lipid metabolism, LCA exposure induced a selective upregulation of *hadh* and *acly* transcript levels. Conversely, DCA treatment resulted in a significant reduction in *pparab*, while T-LCA selectively increased *fasn* mRNA abundance. No significant changes were detected in *acac1*,* acac2*,* nr1h3*,* cpt1b*,* lpl*, or *srebp1* transcript levels under any experimental condition.

**Table 2:** Relative mRNA abundance of genes involved in glucose and lipid metabolism, as well as energy-sensing pathways, in white adipose tissue of rainbow trout 6 h after intragastric administration of 1 mL·100 g^-1^ body weight of distilled water containing either 1% DMSO alone (control) or containing lithocholic acid (LCA, 500 μM), deoxycholic acid (DCA, 1500 μM), taurolithocholic acid (T-LCA, 1000 μM), or taurodeoxycholic acid (T-DCA, 600 μM). RT-qPCR values were normalized to *actb* and *eef1a1* and expressed as fold change relative to the control group. Data are shown as mean ± SEM (n = 8). Statistical differences between treatments and control were evaluated using Student’s t-test and adjusted using the false discovery rate (FDR) procedure, applied independently to each analytical block within the gene expression dataset. Padj < 0.05 was considered statistically significant

		Control	LCA	DCA	T-LCA	T-DCA
Glucose homeostasis	*pfk*	1.00 ± 0.12	3.27 ± 0.92	1.70 ± 0.24*	1.80 ± 0.37	0.97 ± 0.09
	*pklr*	1.00 ± 0.21	3.93 ± 1.39	3.95 ± 0.72*	3.61 ± 1.70	0.98 ± 0.13
	*fbp1*	1.00 ± 0.25	7.33 ± 2.45	4.14 ± 0.76*	1.98 ± 0.68	1.53 ± 0.50
	*pck*	1.00 ± 0.15	4.16 ± 1.71	2.12 ± 0.56	2.75 ± 0.86	0.92 ± 0.16
	*g6pc*	1.00 ± 0.20	17.26 ± 9.15	2.25 ± 0.72	5.95 ± 2.34	0.95 ± 0.41
	*mlxipl*	1.00 ± 0.11	4.80 ± 1.49	1.66 ± 0.38	1.78 ± 0.39	1.05 ± 0.16
Lipid homeostasis	*acly*	1.00 ± 0.18	3.34 ± 0.88*	1.45 ± 0.16	1.37 ± 0.21	0.92 ± 0.18
	*fasn*	1.00 ± 0.18	5.11 ± 1.68	1.54 ± 0.36	2.44 ± 0.42*	1.94 ± 0.53
	*cpt1b*	1.00 ± 0.17	2.88 ± 0.75	1.33 ± 0.23	1.47 ± 0.32	1.20 ± 0.20
	*acac1*	1.00 ± 0.14	3.50 ± 1.13	1.58 ± 0.39	1.50 ± 0.26	1.11 ± 0.19
	*acac2*	1.00 ± 0.10	3.55 ± 0.96	1.32 ± 0.35	1.57 ± 0.40	1.38 ± 0.21
	*hadh*	1.00 ± 0.16	5.97 ± 1.77*	1.98 ± 0.42	1.92 ± 0.51	1.51 ± 0.43
	*lpl*	1.00 ± 0.15	4.70 ± 1.52	0.96 ± 0.21	2.21 ± 0.65	1.21 ± 0.27
	*nr1h3*	1.00 ± 0.09	6.51 ± 2.16	2.54 ± 0.72	3.15 ± 0.80	1.82 ± 0.41
	*pparab*	1.00 ± 0.14	0.80 ± 0.10	0.51 ± 0.08*	0.82 ± 0.06	0.62 ± 0.09
	*srebp1*	1.00 ± 0.13	2.15 ± 0.59	1.09 ± 0.24	1.96 ± 0.58	1.76 ± 0.39
Energy sensors	*prkaa1*	1.00 ± 0.11	1.55 ± 0.16*	1.42 ± 0.24	1.33 ± 0.18	0.99 ± 0.06
	*prkaa2*	1.00 ± 0.10	3.66 ± 1.02*	1.35 ± 0.29	2.29 ± 0.59	1.45 ± 0.19
	*mtor*	1.00 ± 0.10	3.34 ± 0.87*	1.56 ± 0.29	1.81 ± 0.41	1.78 ± 0.34

**Fig. 5:**
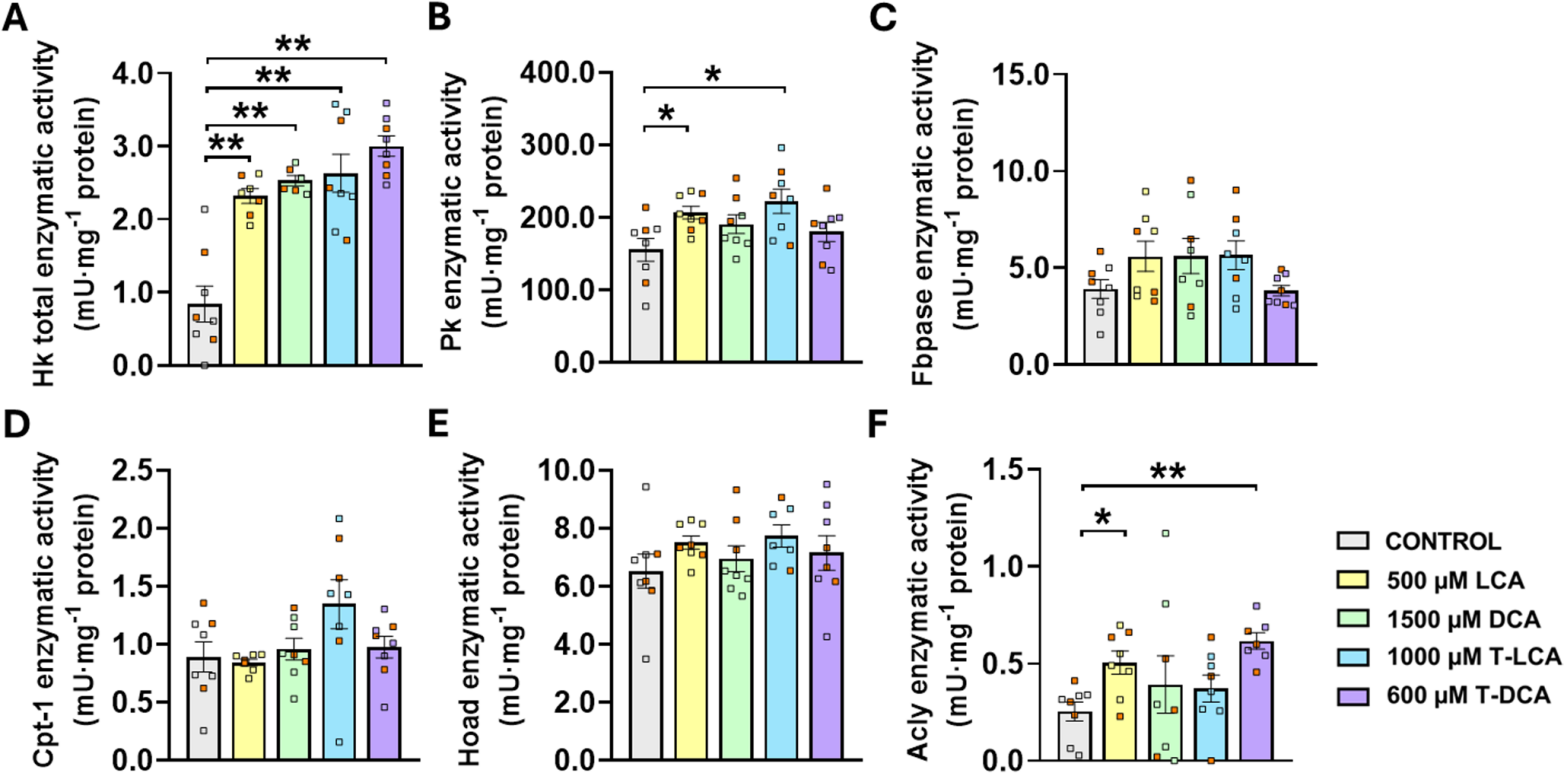
Changes in specific enzymatic activity (mU·mg^− 1^ protein) of key enzymes involved in glucose homeostasis, including (**A**) Total hexokinase, (**B**) pyruvate kinase, (**C**) fructose 1,6-bisphosphatase, and in lipid homeostasis, including (**D**) carnitine palmitoyltransferase 1, (**E**) 3-Hydroxyacyl-CoA dehydrogenase and (**F**) ATP citrate synthase, in white adipose tissue of rainbow trout 6 h after intragastric administration of 1 mL·100 g⁻¹ body weight of distilled water with either 1% DMSO alone (control) or containing 500 µM lithocholic acid (LCA), 1500 µM deoxycholic acid (DCA), 1000 µM taurolithocholic acid (T-LCA) or 600 µM taurodeoxycholic acid (T-DCA). Data are shown as mean ± SEM (*n* = 8). Statistical differences between treatments and control were evaluated using Student’s t-test and adjusted using the false discovery rate; *P_adj_ < 0.05 and **P_adj_ < 0.01. Orange squares indicate the samples originally used for RNA sequencing, which were subsequently integrated into the dataset alongside the remaining samples

The activities of enzymes involved in glucose and lipid metabolism in the analyzed tissue exhibited treatment-specific responses (Fig. 5). LCA administration significantly increased Pk activity (Fig. 5B) associated with glucose metabolism, as well as Acly activity (Fig. 5F) linked to lipid metabolism. An increase in Pk and Acly activities was also observed following T-LCA (Fig. 5B) and T-DCA (Fig. 5F) treatment, respectively. In addition, all treatments induced a strong and significant increase in total Hk activity (Fig. 5A). In contrast, no significant changes were detected in Fbpase (Fig. 5C), Cpt-1 (Fig. 5D) or Hoad (Fig. 5E) activities under any experimental condition.

**Fig. 6:**
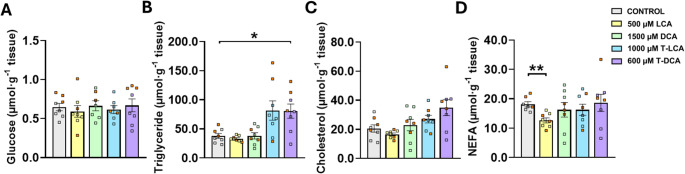
Changes in white adipose tissue metabolite levels, including (**A**) glucose, (**B**) non-esterified fatty acids (NEFA), (**C**) cholesterol, and (**D**) triglyceride in rainbow trout 6 h after intragastric administration of 1 mL·100 g⁻¹ body weight of distilled water with either 1% DMSO alone (control) or containing 500 µM lithocholic acid (LCA), 1500 µM deoxycholic acid (DCA), 1000 µM taurolithocholic acid (T-LCA) or 600 µM taurodeoxycholic acid (T-DCA). Data are shown as mean ± SEM (*n* = 8). Statistical differences between treatments and control were evaluated using Student’s t-test and adjusted using the false discovery rate; *P_adj_ < 0.05 and **P_adj_ < 0.01. Orange squares indicate the samples originally used for RNA sequencing, which were subsequently integrated into the dataset alongside the remaining samples

Metabolite levels in WAT exhibited few treatment-specific alterations in response to SBA administration (Fig. 6). Notably, LCA treatment resulted in a significant decrease in NEFA content (Fig. 6D), whereas T-DCA exposure led to an increase in triglyceride levels (Fig. 6B). In contrast, no significant changes were observed in glucose (Fig. 6A) or cholesterol (Fig. 6C) concentrations under any treatment condition.

## Discussion

BAs are increasingly recognized as signaling molecules exerting direct regulatory functions in several peripheral and central tissues (Perino et al. 2021). In this context, WAT has emerged as a physiologically relevant target of BA action in mammals, where specific BA species directly modulate adipose homeostasis (Schmid et al. 2023). However, whether comparable BA-mediated effects occur in fish adipose tissue remains unknown. Using rainbow trout as a teleost model, the present study therefore examined SBA-induced transcriptional and metabolic responses in WAT. Based on previous evidence of rapid systemic reabsorption of SBAs and early transcriptional responses in physiologically relevant tissues in this species (Pérez-Tierra et al. 2025), WAT was analyzed at an acute post-administration point.

Major results showed that the magnitude and nature of the transcriptional responses in WAT were strongly dependent on the specific SBA administered. LCA and DCA induced the highest number of differentially expressed genes, whereas their taurine-conjugated forms elicited more moderate responses, highlighting the importance of BA chemical structure—and particularly conjugation status—in shaping SBA-mediated effects in adipose tissue. Moreover, the distinct enrichment of KEGG pathways across treatments indicates that individual SBAs activate specific pathways rather than generating a uniform transcriptional response. Although comparable data in fish WAT are currently lacking, this response pattern closely mirrors observations in human and murine adipocytes, where BA identity determines the direction and magnitude of adipose metabolic regulation (Schmid et al. 2019). More broadly, these findings align with the well-established tissue- and BA–specific nature of BA signaling described across target tissues in both mammals (Liu et al. 2014; Song et al. 2015; Zheng et al. 2021) and fish (Wang et al. 2023; Du et al. 2024; Pérez-Tierra et al. 2025, 2026). This parallel supports the concept that rainbow trout WAT retains a differential and compound-selective sensitivity to SBAs, consistent with the broader physiological diversity of bile acid signaling mechanisms among vertebrates.

### Secondary Bile Acid–Mediated Regulation of Glucose Homeostasis in Rainbow Trout Adipose Tissue

Although the liver has traditionally been regarded as the main metabolic organ in fish (Van De Pol et al. 2017), growing evidence indicates that adipose tissue plays an active role in whole-body energy balance, responding dynamically to hormonal and nutritional cues (Weil et al. 2013). In teleosts, WAT extends beyond its classical function as a triacylglycerol storage site and displays substantial expression of genes involved in fatty acid and glucose metabolism, thus contributing to lipid mobilization and systemic energy homeostasis (Weil et al. 2013; Rosell-Moll et al. 2025). Although adipose tissue is not considered a gluconeogenic organ, glucose taken up by adipocytes is mainly channeled into glycolysis and anabolic pathways that can support lipid storage. In this context, salmonid adipocytes have been shown to convert glucose into storage lipids, although with lower capacity than the liver (Bou et al. 2016).

Despite the well-documented limited capacity of teleosts, and rainbow trout in particular, to efficiently utilize dietary carbohydrates (Polakof et al. 2012), glucose metabolism remains physiologically relevant in peripheral tissues. In carnivorous teleosts, this relevance is not reflected by high tissue glucose concentrations but rather by the metabolic use of glucose as a regulatory and anabolic substrate. In this regard, the markedly lower glucose levels in WAT (~ 0.5 µmol g⁻¹ tissue) compared with liver (~ 20 µmol g⁻¹ tissue) (Comesaña et al. 2025; Pérez-Tierra et al. 2026) underscore fundamental differences in glucose handling and functional demand between these organs, where even subtle transcriptional or enzymatic changes may be physiologically meaningful. Within this framework, the present results suggest that BA exposure is associated with modulation of glucose metabolism in rainbow trout WAT in a bile acid-specific manner. Importantly, bile acid identity was also associated with glucose-related responses in the liver under the same experimental conditions (Pérez-Tierra et al. 2026), although clear tissue-specific differences were observed. Thus, in our previous study, LCA was the only BA associated with changes in hepatic glucose metabolism, particularly suggesting a gluconeogenic orientation. In contrast, the absence of coordinated changes in classical gluconeogenic markers, including *pck* and *g6pc* mRNA abundance and Fbpase activity, does not support activation of gluconeogenesis in WAT, in agreement with the view that adipose tissue is not a major gluconeogenic site in vertebrates, including fish (Polakof et al. 2012; Han et al. 2016).

Classical glycolytic transcriptional markers were also unaffected by LCA, but this treatment was associated with increased expression of *prkaa1*, *prkaa2* and *mtor*, together with higher hexokinase and pyruvate kinase activities, suggesting a modulation of cellular energy sensing and glucose utilization rather than transcriptional reprogramming of glycolysis. In contrast, DCA was associated with a more focused transcriptional response involving increased expression of *pfk*, *pklr* and *fbp1*, suggestive of partial adjustment of glycolytic components without evidence of global metabolic reprogramming.

Together, these patterns suggest that SBAs may contribute to fine-tuning of glucose handling in adipose tissue by influencing glycolytic flux and energy-status signaling, thereby potentially contributing to metabolic flexibility rather than glucose production. Interestingly, even though trout bile acid pools are predominantly taurine-conjugated (Yamamoto et al. 2007; Staessen et al. 2021), these forms (T-LCA, T-DCA) showed negligible transcriptional activity, suggesting lower signaling potency under the acute conditions tested.

### Impact of SBAs in Lipid Homeostasis in Rainbow Trout Adipose Tissue

Despite adipose tissue playing a central role in energy homeostasis, the modulatory effects of SBAs on lipid metabolism in fish remain poorly defined. This study provides insight into how individual SBAs are associated with transcriptional and enzymatic lipid pathway changes in rainbow trout adipose tissue. LCA showed the most pronounced association with lipid-related pathways in WAT. The upregulation of genes involved in acetyl-CoA provision and lipid handling, including *acly* and *hadh*, together with consistent upward trends in *acac1*,* srebp1*,* nr1h3*,* cpt1b*,* acac2* and *lpl*, and increased Acly activity, indicates an overall enhancement of lipid metabolic capacity. However, these changes were not accompanied by increases in Cpt-1 or Hoad activities, suggesting that LCA is not strongly associated with enhanced fatty acid β-oxidation under acute conditions. Consistently, LCA was associated with reduced NEFA levels in WAT without changes in triglyceride or cholesterol content. Plasma measurements under the same conditions showed no changes in circulating NEFA, triglycerides, or cholesterol (Pérez-Tierra et al. 2026), supporting the interpretation that SBA exposure primarily affects intracellular lipid handling rather than systemic lipid mobilization. Together, these responses are consistent with increased metabolic flexibility and lipid turnover within adipocytes without evidence of net lipid mobilization. In contrast, DCA was associated with a more restricted molecular response in WAT, characterized by downregulation of *pparab* without changes in other lipid-related genes or enzymatic and metabolite levels. These findings suggest that DCA has limited association with lipid homeostasis regulation in trout WAT under acute conditions. Notably, DCA was also the only SBA associated with increased *fxra1* expression, the only detectable Fxr paralogue in trout WAT. The concurrent changes in *fxra1* and *pparab* prompted examination of potential receptor interactions, although no functional conclusions can be drawn. This pattern contrasts with mammalian liver, where FXR activation is associated with PPARα induction (Pineda Torra et al. 2003). Evidence from fish supports context-dependent FXR–PPARα associations. In trout liver, only DCA was associated with *fxra1* induction without changes in *pparab*, while LCA, T-LCA and T-DCA showed no effects on either receptor (Pérez-Tierra et al. 2026), indicating tissue-specific divergence.

Dietary BA studies in teleosts report highly variable patterns, including *ppara* upregulation with *fxr* downregulation (Yang et al. 2023), concurrent upregulation of both (Zheng et al. 2024), isolated *ppara* induction (Zhou et al. 2018; Jin et al. 2019), or *ppara* repression depending on BA type, particularly with DCA (Du et al. 2024), alongside FXR activation. Collectively, these findings suggest that FXR–PPARα relationships in fish are context-dependent and not yet fully resolved. A similar complexity is observed for FXR–PPARγ interactions. In mammalian WAT, FXR has been associated with inhibition of adipocyte differentiation and modulation of PPARγ activity (Chen et al. 2017), while other studies suggest FXR-linked lipogenic effects via PPARγ-dependent mechanisms (Shinohara and Fujimori 2020). These findings are relevant given conserved features of adipose regulation between mammals and teleosts (Bou et al. 2017). However, in rainbow trout WAT, evaluation of FXR–PPARγ interactions is limited by very low *pparg* expression, which was below detection in this study. This is consistent with trout adipocyte cultures showing minimal *pparg* transcripts (Balbuena-Pecino et al. 2025) and in vivo studies reporting low *pparg* expression during adipocyte differentiation (Riera-Heredia et al. 2022). These observations suggest that Pparγ may play a limited role in mature trout WAT, potentially restricting FXR–PPARγ interactions to specific developmental or physiological contexts.

Regarding taurine-conjugated BAs, T-DCA exposure was associated with triglyceride accumulation in WAT, together with increased Hk and Acly activities, without changes in fatty acid β-oxidation enzymes or circulating lipid and glucose levels. This pattern suggests a shift toward glucose utilization and lipogenic processes favoring lipid retention within adipose tissue. However, the absence of coordinated transcriptional and systemic changes suggests a limited and localized effect rather than strong metabolic regulation. Similarly, T-LCA was associated with minimal molecular responses, with only fasn upregulation. Given the lack of corresponding enzymatic or metabolite changes, this likely reflects a weak or context-dependent response under acute conditions.

Collectively, these findings underscore the specificity of SBA-associated signaling in WAT and indicate that taurine conjugation is associated with reduced metabolic responsiveness compared with unconjugated SBAs. This aligns with structural diversity among bile acids, which share a steroid nucleus but differ in side-chain modifications and conjugation (Hofmann and Hagey 2008; Schmid et al. 2019; Perino et al. 2021). These structural differences are likely to influence receptor affinity and downstream signaling potential in adipocytes. Under the same experimental conditions, taurine-conjugated SBAs induced stronger transcriptional responses in intestine, while effects in liver and brain were limited (Pérez-Tierra et al. 2025, 2026). These observations highlight BA structure- and tissue-dependent responsiveness, particularly in rainbow trout, where over 98% of the BA pool is taurine-conjugated (Yamamoto et al. 2007; Staessen et al. 2021).

### Bile Acid Sensing and Signaling Pathways in Rainbow Trout Adipose Tissue

The molecular landscape of bile acid (BA) metabolism and signaling in teleost white adipose tissue (WAT) remains poorly understood. By examining BA transporters, receptors, BA-synthesizing enzymes, and FXR-associated pathways, this study highlights that rainbow trout WAT is capable of sensing and responding to BAs, extending prior work describing tissue-specific BA transporter distribution across liver, brain, and gastrointestinal tract (GIT) (Murashita et al. 2013, 2014; Pérez-Tierra et al. 2025). In the brain, Ntcp (*slc10a1*) and Ostα isoforms (*slc51a1*,* slc51a2*) predominate, whereas Asbt (*slc10a2*) and Ostβ (*slc51b*) are major transporters in the GIT, particularly in the distal intestine. In WAT, a profile more like liver than intestine was observed. The basolateral transporters Ostα (*slc51a1*) and Ostβ (*slc51b*) were the most abundantly expressed, while Asbt (*slc10a2*), highly expressed in intestinal tissue, was nearly absent in both WAT and liver. Ostα−2 (*slc51a2*) showed low expression in peripheral tissues but higher levels in the hypothalamus. Bsep, previously detected in rainbow trout liver under dietary conditions (Murashita et al. 2018), was also expressed in WAT, consistent with observations in mammalian adipose tissue (Schmid et al. 2019). Overall, this transporter profile suggests that WAT may primarily support BA efflux rather than active uptake, potentially limiting intracellular BA accumulation while allowing signaling capacity. Transcriptomic analyses showed that *slc51a1* and *slc51b* were broadly upregulated across SBAs, whereas *slc10a2* responded only to LCA and remained otherwise minimally expressed, suggesting inducible and context-dependent regulation. qPCR validation confirmed *slc51a1* induction by LCA and DCA, although variability across individuals likely reflects adipose tissue heterogeneity in cellular composition, vascularization, and metabolic state (Esteve Ràfols 2014; Rosell-Moll et al. 2025), which may reduce statistical power in bulk tissue analyses.

FXR signaling in WAT also showed strong tissue- and paralog-specific organization. Among FXR paralogs, fxra1 was the predominant isoform, whereas fxra2, fxrb1, and fxrb2 were not detected. This pattern resembles proximal intestine, where fxra1 also predominates (Pérez-Tierra et al. 2025), and contrasts with liver, where other paralogs are relevant (Pérez-Tierra et al. 2026). Upon SBA exposure, RNA-seq suggested selective responsiveness of FXR paralogs: *fxra1* was associated with DCA exposure, while *fxrb1* responded to DCA and T-DCA. However, only *fxra1* was consistently validated by qPCR, identifying it as the primary BA-responsive FXR paralog in trout WAT. This regulatory pattern is tissue dependent. In intestine, *fxra1* is downregulated by SBAs (Pérez-Tierra et al. 2025), whereas in liver it is upregulated (Pérez-Tierra et al. 2026), mirroring WAT responses. Thus, FXR-associated transcription in trout exhibits BA- and tissue-dependent patterns rather than a unified systemic response. Other FXR downstream components were weakly expressed. Shp and Hnf4α showed very low basal expression in WAT compared with liver (Murashita et al. 2013; Pérez-Tierra et al. 2026). Although RNA-seq suggested potential *hnf4a* induction by DCA, expression levels were near detection limits, preventing robust validation. These findings suggest limited basal engagement of these pathways in WAT under acute conditions. Gpbar1 (TGR5) also showed extremely low expression, often near detection limits, suggesting minimal involvement in basal or acute SBA responses. This contrasts with mammals, where TGR5 in adipose tissue has been linked to regulation of lipid metabolism and energy expenditure (Teodoro et al. 2016; Chen et al. 2017; Velazquez-Villegas et al. 2018; Schmid et al. 2019; Liu et al. 2025).

Overall, WAT displays a selective BA-sensing system dominated by FXR (fxra1), while TGR5, SHP, and HNF4α appear minimally engaged under basal conditions. This configuration partially overlaps with hepatic FXR signaling but differs markedly from intestinal and mammalian adipose systems. A similar pattern of selectivity was observed in BA-synthesizing enzymes. Under basal conditions, *cyp8b1-2* was the most abundantly expressed transcript, followed by *cyp8b1-1*, whereas *cyp7a1-2* showed lower expression and *cyp7a1-1* was not detected. This is consistent with previous reports in rainbow trout (Murashita et al. 2013) and may reflect a role in systemic BA regulation rather than local synthesis. SBA exposure was associated with isoform-specific responses: *cyp8b1-2* responded broadly, whereas *cyp8b1-1* responded selectively to DCA. However, only *cyp8b1-1* was confirmed by qPCR, likely reflecting tissue heterogeneity. In liver, different regulations were observed (Pérez-Tierra et al. 2026). Across teleosts, BA-specific regulation of *cyp8b1* is highly variable (Zheng et al. 2024; Du et al. 2024), suggesting complex regulatory control. *cyp7a1* showed no significant response in WAT, consistent with its role as a long-term regulator of BA homeostasis in fish (Murashita et al. 2013, 2018; Zhu et al. 2018; Du et al. 2024; Zheng et al. 2024; Cocci et al. 2024; Pérez-Tierra et al. 2026).

## Conclusions

This study provides evidence that secondary bile acids (SBAs) are associated with biologically relevant transcriptional and metabolic responses in white adipose tissue (WAT) of teleost fish. In rainbow trout, WAT exhibits compound-specific responsiveness at transcriptomic and enzymatic levels.

Among SBAs, LCA was most strongly associated with changes in energy-sensing pathways and lipid turnover markers, suggesting enhanced metabolic flexibility without evidence of net lipid mobilization. DCA was associated with more limited and selective responses, indicating potential modulation of bile acid-related signaling and partial adjustment of glycolytic pathways rather than broad metabolic reprogramming.

Taurine-conjugated bile acids generally showed weaker associations with metabolic changes, with T-DCA linked to lipid retention and T-LCA showing minimal effects under acute conditions, highlighting the importance of bile acid structure in shaping adipose responsiveness.

Overall, the data supports a model in which bile acid signaling in rainbow trout WAT is characterized by tissue-specific and compound-specific responsiveness rather than a unified FXR-driven program. However, these interpretations are based on acute molecular and metabolic responses and do not establish direct receptor-mediated causality. Future studies using targeted functional approaches will be required to validate the mechanistic pathways involved.

## Supplementary Information

Below is the link to the electronic supplementary material.

Supplementary Material 1 (XLSX 77.6 KB)

Supplementary Material 2 (PDF 1.12 MB)

## Data Availability

Data is available at Zenodo repository [10.5281/zenodo.18714369].
