# Supplementary material for: White Adipose Tissue as a Functional Target of Secondary Bile Acids in Rainbow Trout (Oncorhynchus mykiss)
Source: Mar Biotechnol (NY). 2026 May 9;28(3):77. doi: 10.1007/s10126-026-10622-5 (PMC13157367; doi:10.1007/s10126-026-10622-5)
Supplement: Supplementary file 2 — Supplementary Material 2 (PDF 1.12 MB) [file 10126_2026_10622_MOESM2_ESM.pdf]

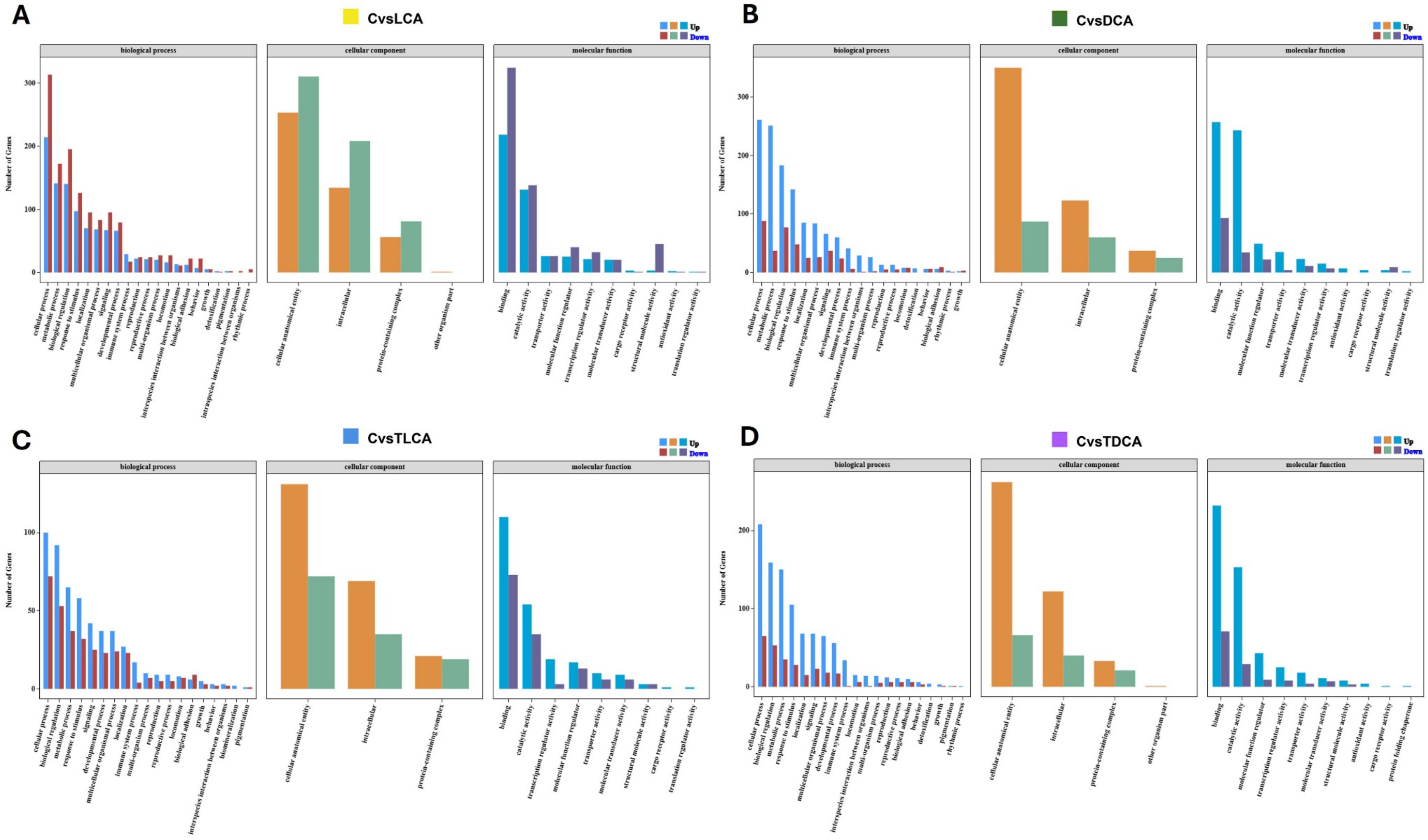

Supplemental Figure S1. Gene Ontology (GO) enrichment analysis of differentially expressed genes in white adipose tissue (WAT) of rainbow trout, comparing control fish with those treated with LCA (A), DCA (B), T-LCA (C), and T-DCA (D). WAT samples were collected 6 h after intragastric administration of 1 mL·100 g^{-1} body weight of either distilled water containing 1% DMSO (control) or solutions of lithocholic acid (LCA, 500 µM), deoxycholic acid (DCA, 1500 µM), taurolithocholic acid (T-LCA, 1000 µM), or taurodeoxycholic acid (T-DCA, 600 µM).
